# Prevalence of Avian Influenza A(H5) and A(H9) Viruses in Live Bird Markets, Bangladesh

**DOI:** 10.3201/eid2412.180879

**Published:** 2018-12

**Authors:** Younjung Kim, Paritosh K. Biswas, Mohammad Giasuddin, Mahmudul Hasan, Rashed Mahmud, Yu-Mei Chang, Steve Essen, Mohammed A. Samad, Nicola S. Lewis, Ian H. Brown, Natalie Moyen, Md. Ahasanul Hoque, Nitish C. Debnath, Dirk U. Pfeiffer, Guillaume Fournié

**Affiliations:** The Royal Veterinary College, Hatfield, UK (Y. Kim, Y.-M. Chang, N. Moyen, D.U. Pfeiffer, G. Fournié);; City University of Hong Kong, Hong Kong, China (Y. Kim, D.U. Pfeiffer);; Chittagong Veterinary and Animal Sciences University, Chittagong, Bangladesh (P.K. Biswas, R. Mahmud, M.A. Hoque);; Bangladesh Livestock Research Institute, Dhaka, Bangladesh (M. Giasuddin, M. Hasan, M.A. Samad);; Animal Health and Plant Agency, Weybridge, UK (S. Essen, N.S. Lewis, I.H. Brown);; Food and Agriculture Organization of the United Nations, Dhaka (N.C. Debnath)

**Keywords:** influenza, influenza virus, avian influenza viruses, viruses, birds, live bird markets, H5 subtype viruses, H9 subtype viruses, Bayesian hierarchical logistic regression, respiratory infections, Bangladesh

## Abstract

We conducted a cross-sectional study in live bird markets (LBMs) in Dhaka and Chittagong, Bangladesh, to estimate the prevalence of avian influenza A(H5) and A(H9) viruses in different types of poultry and environmental areas by using Bayesian hierarchical logistic regression models. We detected these viruses in nearly all LBMs. Prevalence of A(H5) virus was higher in waterfowl than in chickens, whereas prevalence of A(H9) virus was higher in chickens than in waterfowl and, among chicken types, in industrial broilers than in cross-breeds and indigenous breeds. LBMs with >1 wholesaler were more frequently contaminated by A(H5) virus than retail-only LBMs. Prevalence of A(H9) virus in poultry and level of environmental contamination were also higher in LBMs with >1 wholesaler. We found a high level of circulation of both avian influenza viruses in surveyed LBMs. Prevalence was influenced by type of poultry, environmental site, and trading.

Low pathogenicity avian influenza A(H9N2) virus and highly pathogenic avian influenza A(H5N1) virus are endemic in poultry populations in Bangladesh (*1**–**4*). In addition to their adverse effect on poultry production, these viruses have resulted in sporadic influenza cases in humans (*2**,**3*). Because there is potential for generating novel reassortant variants between them or with other virus subtypes, their persistent circulation in poultry poses a serious threat to animal and human health globally (*5**–**9*).

Live bird markets (LBMs) form the backbone of poultry trade in many countries in Asia. Birds of different types and from different geographic areas are introduced daily into LBMs and might be caged together, promoting local transmission of multiple virus subtypes and generating opportunities for reassortment (*10**–**12*). Surveys and routine surveillance have described the abundance and diversity of avian influenza A viruses (AIVs) in LBMs in AIV-endemic countries, including Bangladesh (*1**,**4**,**11**,**13**–**21*). However, only the proportion of positive samples is usually reported, without accounting for the hierarchical data structure, especially the clustering of sampled poultry per LBM. Therefore, a robust assessment of AIV prevalence in LBMs is lacking, although this knowledge is essential to understand AIV epidemiology and optimize surveillance design.

Multiple poultry species and, for each poultry species, multiple breeds are offered for sale in LBMs in Bangladesh. Desi, Sonali, and broiler are the most commonly traded chicken types. Desi, which means “local” in Bengali, are indigenous chicken breeds raised in backyard farms. Sonali is a cross-breed of the Rhode Island Red cocks and Fayoumi hens. Broilers are industrial white-feathered breeds. In addition to varying levels of susceptibility, different poultry types might be raised in different farming systems and traded through different value chains (i.e., the range of activities that businesses perform to deliver products to customers), therefore being exposed to different pathogens and pathogen loads (*22**,**23*). However, the proportion of AIV-positive samples is generally reported as an overall estimate or stratified only by poultry species. Likewise, the relative position of individual LBMs in a regional or national live poultry trading network might also affect AIV prevalence; the sources from which poultry are supplied to traders and the time they spend in LBMs influence the likelihood of virus introduction and amplification in LBMs and vary depending on traders being wholesalers or retailers (*12**,**24*). However, such information is generally poorly documented, or even ignored.

To address these issues, we conducted a cross-sectional survey in the 2 largest cities in Bangladesh, Dhaka and Chittagong, during February–March 2016. First, we estimated prevalence of influenza A(H5) and A(H9) viruses in marketed poultry and the LBM environment. We also accounted for the clustering effect at LBM level by using Bayesian hierarchical logistic regression models. Second, we assessed the effect of type of poultry and environmental site, and the position of LBMs in the poultry value chain on AIV prevalence.

## Materials and Methods

### Sample Collection

An LBM was defined as an open space in which >2 poultry stalls sell live poultry at least once a week, and only those selling >400 poultry/day were considered eligible for this study. We aimed to sample 40 LBMs, and from each of these LBMs, 60 birds and 50 environmental sites (sample size calculations in Technical Appendix 1. We used a stratified cluster sampling design. For poultry, LBMs, stalls within selected LBMs, and birds within selected stalls constituted the primary, secondary, and tertiary sampling units, respectively. For environmental sites, LBMs constituted primary sampling units and environmental sites within selected LBMs constituted secondary sampling units.

We stratified LBMs by city for Dhaka and Chittagong and, within each city, by poultry sales into large and small LBMs, hypothesizing that the risk for AIV infection varies between geographic locations and the number of poultry traded. Also, simple random sampling with too small a sample size of LBMs was not likely to capture diversity of LBM types because the distribution of LBMs as a function of their size tended to be right-skewed; the largest LBMs were often wholesale markets (*24**,**25*). We hypothesized that samples of different origins have different AIV prevalences and thus stratified birds and environmental sites into 5 types of poultry and 10 types of environmental sites commonly found to be contaminated with AIV (*26*) (Technical Appendix 2 Tables 1, 2).

At the first sampling stage, we sampled 40 LBMs. The number of LBMs selected in Dhaka (n = 26) and Chittagong (n = 14) was proportional to the number of LBMs eligible in each city (n = 80 for Dhaka and n = 36 for Chittagong). In each city, we further stratified LBMs by size: 50% of the selected LBMs were large, trading the highest number of poultry (13 largest LBMs in Dhaka and 7 largest LBMs in Chittagong); 50% were small, randomly selected from the bottom 50% of eligible LBMs in terms of number of poultry traded.

At the second sampling stage, we randomly selected stalls and environmental sites in each LBM independently for each type of poultry and environmental site. We created a list of stalls selling each poultry type for each LBM. We then selected stalls from these lists by using a random number generator. Likewise, for each type of environmental site, we selected sites from a list of sites identified in each LBM by using a random number generator. We collected 1 swab specimen from each environmental site. We pooled 5 swab specimens collected from the same LBM and site type.

At the third sampling stage, for each poultry type, we randomly selected 5 birds from each of the stalls selected for that type and collected cloacal and oropharyngeal swab specimens from each of the selected birds. We pooled 5 swab specimens collected from the same stall and poultry type separately for cloacal and oropharyngeal swab specimens. We transported samples collected in Chittagong on the day of sampling to the Chittagong Veterinary and Animal Sciences University (Chittagong) and samples collected in Dhaka on the day of sampling to the Bangladesh Livestock Research Institute (Dhaka). Samples were stored at −80°C until diagnostic laboratory processing.

### Sample Screening

We screened pools for AIVs by using a real-time reverse transcription PCR (RT-PCR) and specific primers and probes (*27**,**28*). We extracted virus RNA by using the MagMAX RNA Isolation Kit (QIAGEN, Hilden, Germany) and reverse transcribed and amplified virus RNA by using the AgPath-ID One-Step RT-PCR (ThermoFisher Scientific, Waltham, MA, USA). We then screened a pool with a cycle threshold (C_t_) <40 for the AIV matrix gene for the H5 and H9 genes. Results were considered positive for the H5 subtype if C_t_ <38 and positive for the H9 subtype if C_t_<40 (*27**,**28*). A pool was considered positive for AIV if its C_t_ for the AIV matrix gene <38 or if it was positive for any of the H5 and H9 subtypes. A given group of 5 birds was considered positive if any of its cloacal and oropharyngeal pools showed a positive result.

### Bayesian Hierarchical Logistic Regression Models

We developed 2-level Bayesian hierarchical logistic regression models to estimate LBM-level, bird-level, and environmental swab specimen–level prevalence from pooled swab samples, accounting for lower-level (swab specimens) and higher-level (LBMs) risk factors. We developed separate models for poultry and environmental samples to avoid parameters related to different sampling units interfering with each other (*29*). We used LBM type (retail or mixed), city (Chittagong or Dhaka), and size (small or large) as LBM-level risk factors. We defined an LBM with only retailers (i.e., trader selling poultry to end-users only) as retail and an LBM with >1 wholesaler (i.e., trader selling poultry to other traders) as mixed. Each LBM-level risk factor (LBM type, city, and size) was assessed separately because we could not include them simultaneously in any given model due to the small number of sampled LBMs. In models for poultry samples, we differentiated birds into 1) chicken and waterfowl or 2) broiler, Desi, Sonali (i.e., chicken types), and waterfowl. Waterfowl were not differentiated further because of the small number of pools collected from ducks and geese. In models for environmental samples, we differentiated environmental sites into stall and slaughter areas or classified as environmental area without differentiation. We ran models (Technical Appendix 1) by using a Markov Chain Monte Carlo simulation in JAGS (*30*) and R.3.4.2 (*31*).

## Results

### Descriptive Results for Pooled Swab Samples

We collected 477 pairs of cloacal and oropharyngeal pooled samples from 2,384 birds, and 400 environmental pooled samples from 2,000 environmental sites in 40 LBMs in Chittagong and Dhaka. Each pool was composed of 5 swab specimens, except for 1 pair of cloacal and oropharyngeal pools made from 4 swab specimens collected from geese. We collected 12 pairs of cloacal and oropharyngeal pooled samples from all LBMs, except for 11 pairs from 3 LBMs. We sampled chickens in all LBMs (8–12 pairs/LBM), and waterfowl in 25 LBMs (0–4 pairs/LBM). Broilers accounted for most samples (32.1%), followed by Desi (26.6%) and Sonali (25.6%). Ducks accounted for 76% of 75 pool pairs collected from waterfowl and geese accounted for 24%. We collected 10 environmental pools in each LBM (stall areas: 4–8 pools, slaughter areas, 2–6 pools).

Of 47.4% (416/877) pools considered positive for AIV, 6.5% pools were negative for the AIV matrix gene but positive for any of the H9 and H5 subtypes. The H9 subtype (63.2% positive pools) was detected more frequently than the H5 subtype (21.6%), and 12.3% of pools were positive for both subtypes and 27.4% of pools were negative for both subtypes. Although 80.0% of the LBMs had *>*1 A(H5) virus–positive poultry or environmental pool, 97.5% had >1 A(H9) virus–positive poultry or environmental pool. We determined the prevalence of pools that were positive for A(H5) and A(H9) viruses according to sample and LBM type (Table 1).

**Table 1:** Prevalence of avian influenza A(H5) and A(H9) viruses in pooled poultry and environmental samples, Chittagong and Dhaka, Bangladesh*

Sample type	No. pools	A(H5) virus prevalence, %			A(H9) virus prevalence, %	
Observed pool level	Estimated bird level (95% HDI)†	Observed pool level	Estimated bird level (95% HDI)†
Poultry						
Retail LBM						
Broiler	96	5.2	0.9 (0–9.4)		37.5	10.8 (2.3–22.5)
Sonali	62	3.2	1.4 (0–13.2)		32.3	6.6 (1.2–14.5)
Desi	61	3.4	1.3 (0–12.2)		29.5	6.8 (1.3–14.9)
Waterfowl	20	50.0	8.1 (0–46.8)		25.0	2.8 (0.3–7.0)
Mixed LBM						
Broiler	57	1.8	0.9 (0–4.0)		47.4	13.1 (1.2–30.1)
Sonali	60	10.0	1.4 (0–5.7)		31.7	8.0 (0.4–19.8)
Desi	66	9.1	1.3 (0–5.2)		39.4	8.3 (0.5–20.4)
Waterfowl	55	27.3	7.6 (0–24.6)		16.4	3.4 (0.1–9.7)
Environmental site						
Retail LBM						
Stall area	101	5.9	1.5 (0–10.4)		16.8	3.2 (0.1–9.1)
Slaughter area	99	7.1	1.4 (0–10.1)		25.3	6.2 (0.2–16.6)
Mixed LBM						
Stall area	102	15.7	3.1 (0–11.3)		23.5	5.2 (0.1–14.1)
Slaughter area	98	14.3	3.0 (0–11.0)		37.8	9.9 (0.4–25.0)

*Desi, which means “local” in Bengali, are indigenous chicken breeds raised in backyard farms. Sonali is a cross-breed of the Rhode Island Red cocks and Fayoumi hens. HDI, high-density interval; LBM, live bird market. †Bird and environmental swab specimen–level prevalence in contaminated live bird markets. Median values are reported.

Approximately 33.3% of pools collected from waterfowl were positive for A(H5) virus, whereas only 5.5% of those collected from chickens were positive. In contrast, the prevalence of A(H9) virus–positive pools was higher in chickens (36.3%) than in waterfowl (18.7%). Among waterfowl, ducks (19.3%) and geese (16.7%) had a similar prevalence of A(H9) virus–positive pools, but the prevalence of A(H5) virus–positive pools was higher in ducks (36.8%) than in geese (22.2%). For both H5 and H9 subtypes, the prevalence of positive pools was higher for oropharyngeal samples (8.6% for H5 and 31.9% for H9) than for cloacal samples (3.6% and 9.9%) in all surveyed poultry types (Technical Appendix 2 Table 1). 

Approximately 25% of environmental pools were positive for A(H9) virus, and the prevalence of positive pools was higher in slaughter areas (31.5%), especially knives and boards used for slaughter and processing, than stall areas (20.2%). The prevalence of A(H5) virus–positive environmental pools was lower (10.8%) and did not vary between slaughter and stall areas (Technical Appendix 2 Table 2).

### Bayesian Model Results

Convergence was achieved for all models; the Gelman and Rubin statistic was <1.001 and the effective sample size was >10,000 for all parameters. For each AIV subtype, the best models reasonably predicted the number of positive pools (Technical Appendix 2 Figure 1). In the best H5 models (i.e., lowest deviance information criterion), A(H5) virus prevalence differed according to poultry species (chicken, waterfowl), but not according to the type of environmental site. In contrast, in the best H9 models, A(H9) virus prevalence differed according to type of poultry (broiler, Desi, Sonali, waterfowl) and environmental site (slaughter and stall area). For both subtypes, LBM size and city did not improve model fit when compared with LBM type. For ease of comparison between the 2 AIV subtypes, we report LBM-level, bird-level, and environmental swab specimen–level prevalences of A(H5) and A(H9) viruses on the basis of the best H9 models with LBM type (Tables 1, 2). This reporting did not affect interpretation of results, and we provide estimates obtained with more parsimonious models (Technical Appendix 2 Tables 3–6).

**Table 2:** Prevalence of avian influenza A(H5) and A(H9) viruses in LBMs, Chittagong and Dhaka, Bangladesh*

Sample type	No. LBMs	H5 virus median prevalence, % (95% HDI)	H9 virus median prevalence, % (95% HDI)
Poultry			
Retail LBM	20	69.9 *(*40.2–100.0)	96.4 (85.5–100.0)
Mixed LBM	20	92.0 (72.3–100.0)	96.0 (84.0–100.0)
Environmental sites			
Retail LBM	20	76.5 (47.2–100.0)	94.9 (80.5–100.0)
Mixed LBM	20	93.2 (75.5–100.0)	96.0 (84.0–100.0)

*Prevalence estimates were made by using the best models. HDI, high-density interval; LBM, live bird market.

LBM-level A(H5) virus prevalence was lower in retail LBMs than in mixed LBMs, and the posterior median estimate was ≈100% for mixed LBMs. However, among contaminated LBMs, levels of virus detection in birds and environmental areas did not vary between LBM types, but A(H5) virus prevalence in waterfowl was ≈6 times higher than in chickens (Figure). The prevalence did not vary between chicken breeds or environmental areas.

**Figure:**
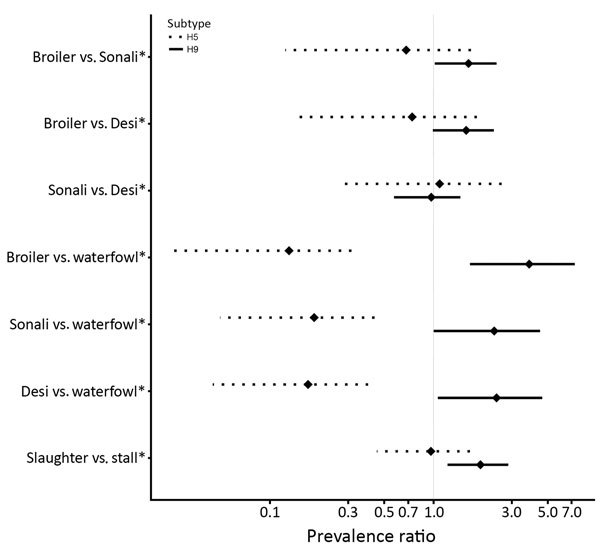
Bird and environmental swab specimen–level avian influenza A(H5) and A(H9) virus prevalence ratios, Bangladesh. Dotted lines indicate H5 subtypes, and solid lines indicate H9 subtypes. Diamonds indicate median values, and horizontal bars indicate 95% high-density interval of a given prevalence ratio. Asterisks (*) indicate reference groups for each comparison. Desi, which means “local” in Bengali, are indigenous chicken breeds raised in backyard farms. Sonali is a cross-breed of the Rhode Island Red cocks and Fayoumi hens.

In contrast to that for A(H5) virus, we found that the posterior median estimate of the LBM-level A(H9) virus prevalence was ≈100% for retail and mixed LBM groups, but the level of virus detection in birds and environmental areas was higher for mixed LBMs than for retail LBMs. A(H9) virus prevalence was highest in broilers and lowest in waterfowl. The prevalence in broilers was 3.8 times as high as that in waterfowl and 1.6 times as high as that in Desi and Sonali (Figure). The environmental swab specimen–level prevalence was ≈2 times as high for slaughter areas than for stall areas (Figure).

## Discussion

We detected A(H5) and A(H9) viruses in marketed poultry and environmental sites in nearly all LBMs sampled in Chittagong and Dhaka. The prevalence of A(H5) virus was higher in waterfowl than in chickens, whereas the prevalence of A(H9) virus was higher in chickens than waterfowl and also varied among chicken types, being more prevalent in broilers than in Desi and Sonali local breeds. Slaughter areas were more frequently contaminated by A(H9) virus than stall areas. Whereas mixed LBMs were more frequently contaminated by A(H5) virus than were retail LBMs, prevalence of A(H9) virus was higher in mixed LBMs than in retail LBMs for birds and environmental areas.

AIVs were ubiquitous in surveyed LBMs. The LBM-level prevalence of A(H5) virus in Bangladesh was higher than in other AIV-endemic countries, including Egypt (*32*) and Vietnam (*16*). For both AIV subtypes, LBM-level prevalence was also higher than in another study conducted in Chittagong (*21*), which found that 17.5% of LBMs had >1 environmental sample pool contaminated by A(H5) virus and 12.5% of LBMs had >1 environmental sample pool contaminated by A(H9) virus. This difference might have been caused by different sampling schemes, in our study, we collected a larger number of pools per LBM.

Bird-level prevalence was also higher than that reported in other AIV-endemic countries, including Bangladesh (*1**,**4**,**16**,**19*). However, care must be taken when comparing these results because studies used different study designs and sample screening protocols over different periods. Bird-level prevalence for contaminated LBMs was much higher than for virologic surveys conducted in backyard and commercial farms in Bangladesh (*1**,**4**,**33**,**34*). This finding suggests that virus transmission was amplified along the value chain from farms to LBMs. Overcrowding and continuous supply of susceptible birds of different species and breeds might have created conditions promoting the silent transmission of AIVs within these markets (*10*).

Our results suggest that birds in LBMs with a mixture of wholesalers and retailers were at higher risk for infection than birds in LBMs with primarily retail poultry businesses. Poultry value chains supplying different business types might differ structurally, thereby affecting the risk for introduced birds being already infected. Wholesalers generally trade a larger number of birds from more diverse geographic origins than do retailers (*23*) and therefore might have increased likelihood of virus introduction into mixed LBMs. Moreover, because wholesalers might sell birds to retailers in the same LBM (*23*), virus amplification might be increased through the presence of wholesalers.

The higher prevalence of A(H9) virus in broilers than in Sonali and Desi might result from differences in the structure of their respective value chains (*23*). Depending on the chicken type, different value chain actors might be involved and their trading practices might differ (*23*). The amount of time chickens spend with traders, the density at which chickens are kept in flocks of traders, and the frequency of contact with chickens from other flocks might vary with chicken type. The greater number of broilers marketed in surveyed LBMs might mean that broilers are more likely than Desi and Sonali to be sourced from large numbers of flocks, which are then mixed in densely populated trucks during transport to LBMs, promoting AIV transmission. However, these prevalence patterns might also be caused by varying levels of genetic susceptibility to AIV infection (*35**,**36*). Further investigations are needed to disentangle the possible influences of trade-related and genetic factors on AIV transmission in these chicken types. The higher level of contamination with A(H9) virus in slaughter areas than in stall areas suggests that, in the absence of appropriate biosecurity measures, slaughtering is likely to expose humans to AIVs by fomite transmission (*37*).

Co-circulation of A(H5) and A(H9) viruses arouses concerns over evolution of novel reassortant variants (*5**–**8*). Detection of both subtypes in some poultry pools suggests that these subtypes co-circulated near each other or in the same host during the study period. Although A(H5) viruses have considerable variability in their ability to infect, cause disease, and be transmitted among waterfowl (*38*), waterfowl are generally known to be less susceptible to highly pathogenic avian influenza A(H5N1) viruses (*39*). Therefore, waterfowl could harbor this virus but remain asymptomatic and serve as a potential host for genesis of novel AIVs in the presence of other virus subtypes. Also, the high level of A(H9) virus circulation among chickens could provide an ideal environment for virus diversification and selection in the LBM system. The different prevalence patterns in chickens and waterfowl observed suggest that these poultry species should be separated in LBMs and that active surveillance of novel reassortant variants should be implemented.

This study had some limitations. First, our models only accounted for clustering of sampled birds at the LBM level, but not at stall level. It is plausible that clustering of samples at stall level has less influence on AIV infection probability across the study population than clustering at the LBM level because stallholders in a given LBM in Bangladesh are likely to be supplied by the same traders and trade between each another (*23*). However, potential risk factors at stall level, such as ducks and hygiene level (*21*), might cause heterogeneous levels of AIV infection across stalls.

Second, our models did not account for the fact that sampling units in each stratum were selected with unequal probabilities. Although we selected different numbers of birds for each poultry type to account for variations in poultry populations, birds were still selected with different probabilities because their populations varied between clusters and strata. This selection might have resulted in larger SEs and thus less precise estimates compared with what could have been obtained with proportional sample sizes. Moreover, the overall prevalence might have been biased toward prevalence in samples selected with higher probabilities.

Third, our models assumed perfect sensitivity and specificity of real-time RT-PCR for pooled samples. The assays used in this study are considered highly sensitive and specific (*27**,**28*), and previous studies did not report any differences in virus detection for pooled and individual samples (*40**–**42*). Furthermore, virus detection in our study was based on parallel interpretation of cloacal or oropharyngeal sample test results (i.e., positive if >1 was positive). However, pools that were negative for the AIV matrix gene but positive for any of the H5 and H9 subtypes indicate that accounting for actual test sensitivity and specificity would enable more robust prevalence estimation. Virus isolation might be attempted for RT-PCR–positive pools to assess the viability of virus material. However, this testing was not attempted in our study. Each pool consisted of swab specimens from different birds or environmental sites. Thus, multiple AIV subtypes and virus species, including Newcastle disease viruses, could be present in the same pool and interfere with growth of each virus in chicken eggs (*43*). Should such studies be replicated, the collection of individual swab specimens and their pooling at the laboratory is recommended to enable analysis of individual swab specimens that formed a virus-positive pool.

Fourth, we collected samples over a short period to reduce variability that could arise from seasonal variations in AIV prevalence. We focused on winter months, which are often reported to be periods of higher risk for AIV infection (*44*). Therefore, our estimates only represented AIV prevalence during that period and did not capture seasonal changes.

Contrary to previous cross-sectional studies, our approach enabled us to estimate AIV prevalence not only by poultry species but also by chicken type and account for the type of LBMs in which sampled poultry were marketed. Despite most AIV surveys and surveillance activities being based on multistage sampling, single-level analytic methods are generally used to analyze their results, while ignoring within-market correlation in poultry infection status. Accounting for this effect by incorporating LBM-specific random effects in a hierarchical model, and enabling mutual influence between bird-level, environmental swab specimen–level, and LBM-level parameters, improved the reliability of prevalence estimates (*29*). When applied to other settings, this approach needs to be adapted on the basis of an understanding of the variety of poultry value chains. Information about LBM locations and about trading practices and numbers and types of poultry sold within these LBMs is rarely readily available and would need to be collected to inform the study design.

In conclusion, LBMs surveyed in Bangladesh were highly contaminated by A(H5) and A(H9) viruses. The level of virus detection was associated with the type of poultry and environmental area and the presence of wholesalers in LBMs. These findings need to be included in the design of risk-based surveillance and control interventions aimed at reducing AIV prevalence, human exposure, and the risk for emergence of novel virus reassortant variants.

Technical Appendix 1 Additional information (model calculations) on prevalence of avian influenza A H5 and H9 viruses in live bird markets, Bangladesh.

Technical Appendix 2 Additional information (prevalence data) on prevalence of avian influenza A H5 and H9 viruses in live bird markets, Bangladesh.
