# Supplementary material for: Prevalence of Avian Influenza A(H5) and A(H9) Viruses in Live Bird Markets, Bangladesh
Source: Emerg Infect Dis. 2018 Dec;24(12):2309–16. doi: 10.3201/eid2412.180879 (PMC6256373; doi:10.3201/eid2412.180879)
Supplement: Technical Appendix 2 — Additional information (prevalence data) on prevalence of avian influenza A H5 and H9 viruses in live bird markets, Bangladesh. [file 18-0879-Techapp-s2.pdf]

Article DOI: https://doi.org/10.3201/eid2412.180879

Prevalence of Avian Influenza A(H5) and A(H9) Viruses in Live Bird Markets, Bangladesh

Technical Appendix 2.

Technical Appendix 2 Table 1. Prevalence of avian influenza A(H5) and A(H9) viruses in poultry samples from live bird markets, Bangladesh*

		Pool-level H5 virus prevalence, %			Pool-level H9 virus prevalence, %		
Sample	No. pools†	Cloacal	Oropharyngeal	Combined‡	Cloacal	Oropharyngeal	Combined‡
Chicken							
Broiler	153	0.7	3.3	3.9	13.1	39.2	41.2
Sonali	122	2.5	6.6	6.6	9.8	29.2	32.0
Desi	127	2.4	5.5	6.3	7.1	33.9	34.6
Subtotal	402	1.7	5.0	5.5	10.2	34.6	36.3
Waterfowl							
Duck	57	14.0	29.8	36.8	8.8	17.5	19.3
Goose	18	11.1	22.2	22.2	5.6	16.7	16.7
Subtotal	75	13.3	28.0	33.3	8.0	17.3	18.7
Total	477	3.6	8.6	10.3	9.9	31.9	30.0

*Desi, “local” in Bengali, are indigenous chicken breeds raised in backyard farms. Sonali is a cross-breed of the Rhode Island Red cocks and Fayoumi hens.
†When a given type of poultry was not available, other types of poultry were sampled. All pools contained 5 swab specimens, except for 1 pair of cloacal and oropharyngeal pools from geese, which contained 4 swab specimens.
‡A pool was considered positive if any of its cloacal and oropharyngeal pools tested positive results.

Technical Appendix 2 Table 2. Prevalence of avian influenza A(H5) and A(H9) viruses in environmental samples from live bird markets, Bangladesh

Sample	No. pools*	Pool-level H5 virus prevalence, %	Pool-level H9 virus prevalence, %
Stall area†			
Water run-off	34	8.8	8.8
Poultry cage floor	40	15.0	22.5
Poultry display table	40	10.0	22.5
Poultry drinking water	40	5.0	27.5
Poultry waste disposal area/bin	36	19.4	22.2
Floor in the area where poultry are kept	13	0.0	7.7
Subtotal	203	10.8	20.2
Slaughtering area			
Water run-off	40	10.0	35.0
Floor of slaughtering area	40	12.5	25.0
Poultry waste disposal area/bin	37	10.8	27.0
Chopping and slaughtering table	40	10.0	32.5
Slaughtering and processing knives/board	40	10.0	37.5
Subtotal	197	10.7	31.5
Total	400	10.8	25.8

*When a given type of environmental site was not available, other types of environmental site were sampled.
†In each live bird market, 5 of 6 environmental sites were sampled depending on their availability.

Technical Appendix 2 Table 3. LBM-level prevalence of avian influenza A(H5) virus estimated from best H5 models, Bangladesh*

Sample type	No. LBMs	Median prevalence, % (95% HDI)
Poultry	40	88.7 (69.1–100.0)
Environmental	40	91.9 (74.7–100.0)

*HDI, high-density interval; LBM, live bird market.

Technical Appendix 2 Table 4. Pool-, bird-, and environmental swab specimen–level prevalence of avian influenza A(H5) virus estimated from best H5 models, Bangladesh*

Sample	No. pools	Pool-level prevalence, %	Bird-level median prevalence, % (95% HDI)†
Poultry			
Chicken	402	5.5	1.5 (0–4.0)
Waterfowl	75	33.3	8.9 (0.2–22.1)
Environmental			
Area	400	12.0	2.6 (0.1–6.9)

*HDI, high-density interval.
†Bird- and environmental swab specimen–level prevalence in contaminated live bird markets from the best H5 models.

Technical Appendix 2 Table 5. LBM-level prevalence of avian influenza A(H9) virus estimated from best H9 models, Bangladesh*

Sample type	No. LBMs	Median prevalence, % (95% HDI)
Poultry	40	98.0 (91.8–100.0)
Environmental	40	97.6 (90.3–100.0)

*HDI, high-density interval; LBM, live bird market.

Technical Appendix 2 Table 6. Pool-, bird-, and environmental swab specimen–level prevalence of avian influenza A(H9) virus estimated from best H9 models, Bangladesh*

Sample	No. pools	Pool-level prevalence, %	Bird-level median prevalence (95% HDI)†
Poultry			
Chicken	153	41.2	12.0 (4.8–21.1)
Sonali	122	32.0	7.3 (2.7–13.5)
Desi	127	34.6	7.6 (2.8–13.9)
Waterfowl	75	18.7	3.1 (0.8–6.6)
Environmental			
Stall area	203	20.8	4.0 (1.0–8.7)
Slaughtering area	197	30.5	7.7 (2.1–15.8)

*HDI, high-density interval. Desi, "local" in Bengali, are indigenous chicken breeds raised in backyard farms. Sonali is a cross-breed of the Rhode Island Red cocks and Fayoumi hens.
†Bird- and environmental swab specimen–level prevalence in contaminated live bird markets from the best H9 models.

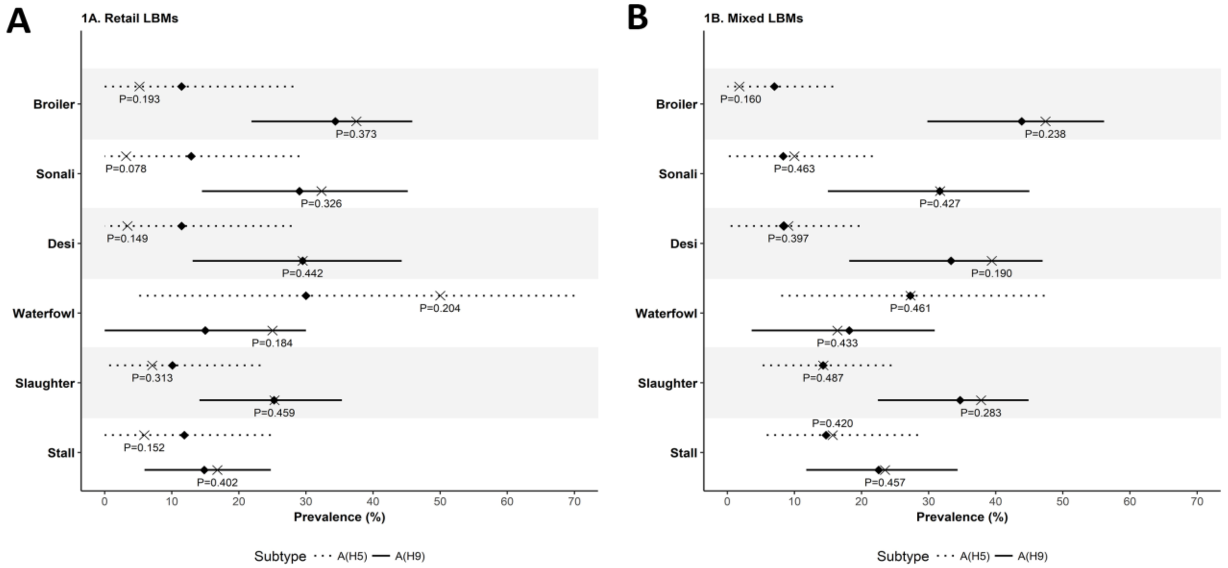

Technical Appendix 2 Figure 1. Posterior predictive checks of the models presented for analysis of prevalence of avian influenza A(H5) and A(H9) viruses in live bird markets, Bangladesh. A) Retail live bird

markets; B) mixed live bird markets. Dotted lines indicate H5 subtypes, and solid lines indicate H9 subtypes. Diamonds indicate median values, horizontal bars indicate 95% high-density interval of the posterior predictive distribution, and ×s indicate observed pool-level prevalences. p values correspond to the proportion of posterior predictive values that are equal to or more extreme than the observed prevalence. Desi, “local” in Bengali, are indigenous chicken breeds raised in backyard farms. Sonali is a cross-breed of the Rhode Island Red cocks and Fayoumi hens.

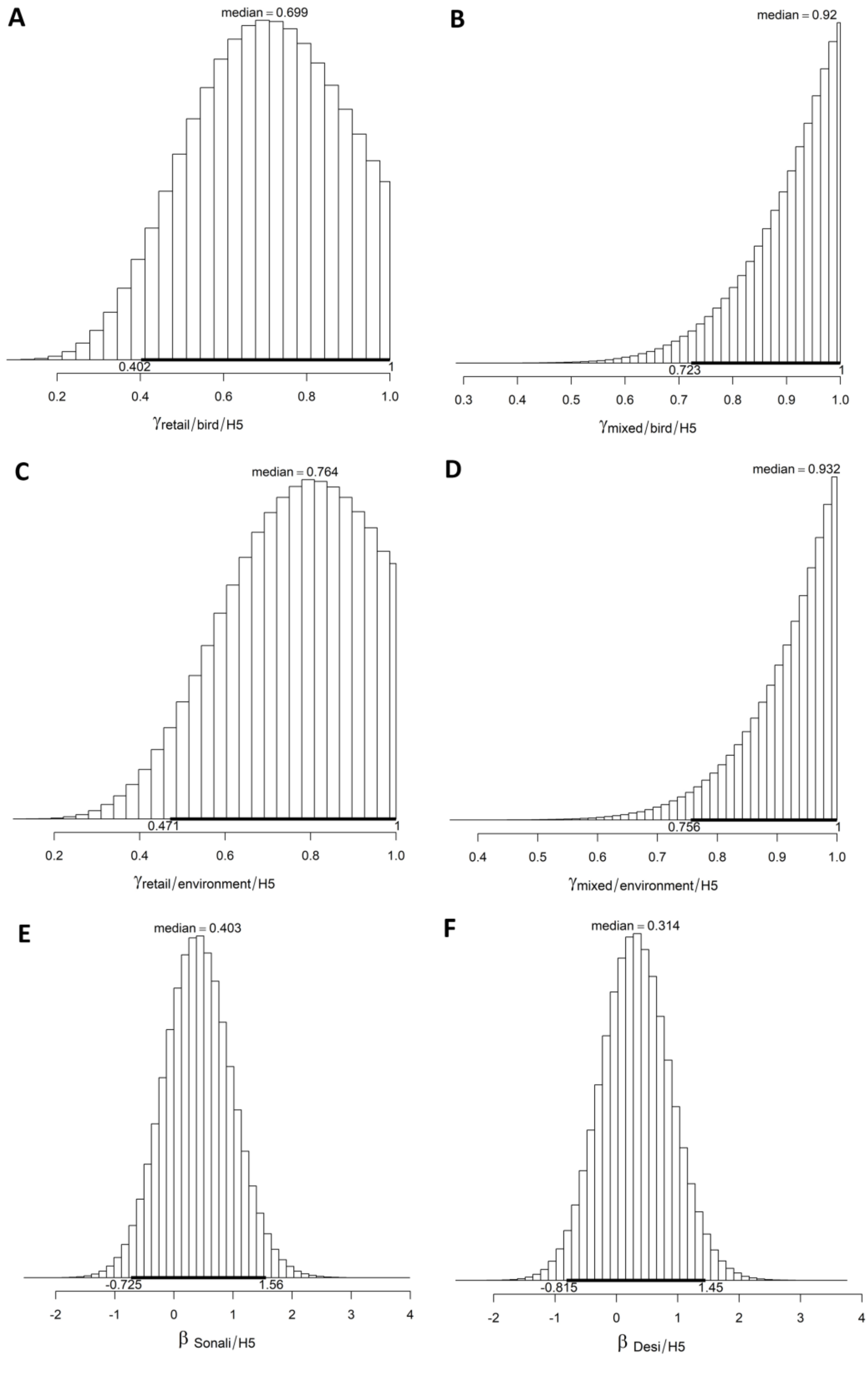
A
median = 0.699
0.402
1
0.2 0.4 0.6 0.8 1.0
γretail/bird/H5
B
median = 0.92
0.723
1
0.3 0.4 0.5 0.6 0.7 0.8 0.9 1.0
γmixed/bird/H5
C
median = 0.764
0.471
1
0.2 0.4 0.6 0.8 1.0
γretail/environment/H5
D
median = 0.932
0.756
1
0.4 0.5 0.6 0.7 0.8 0.9 1.0
γmixed/environment/H5
E
median = 0.403
-0.725
1.56
-2 -1 0 1 2 3 4
β Sonali/H5
F
median = 0.314
-0.815
1.45
-2 -1 0 1 2 3 4
β Desi/H5

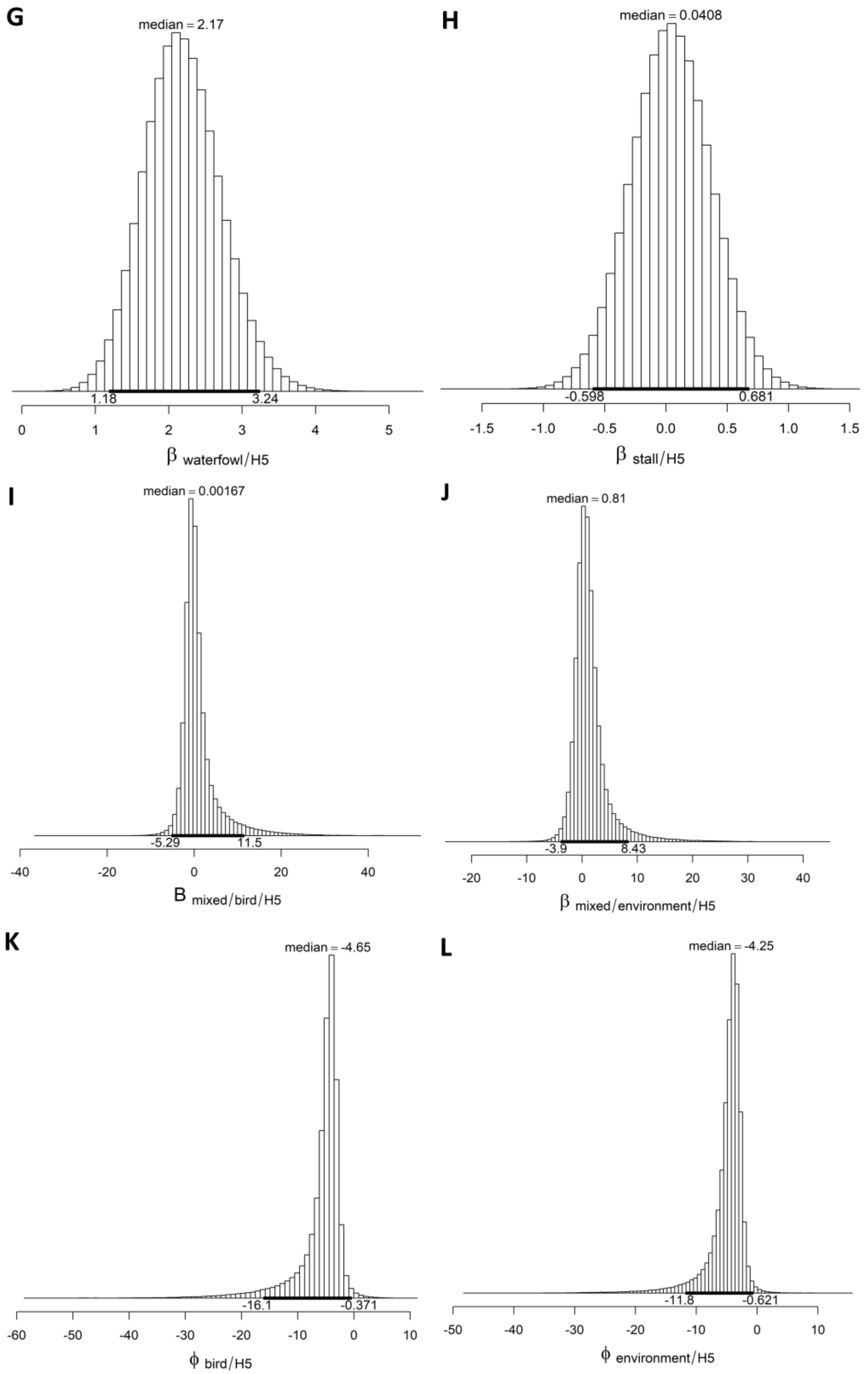

Technical Appendix 2 Figure 2. Posterior distribution of parameters used in models for prevalence of avian influenza A(H5) subtype virus in live bird markets, Bangladesh. Desi, “local” in Bengali, are indigenous chicken breeds raised in backyard farms. Sonali is a cross-breed of the Rhode Island Red

cocks and Fayoumi hens. Values along baselines are medians. Solid horizontal bars indicate 95% high density intervals. Each panel (A–L) shows a different situation that is listed at the bottom of each figure panel. A) γ/retail/bird/H5; B) γ/mixedl/bird/H5; C) γ/retail/environment/H5; D) γ/mixed/environment/H5; E) β/Sonalii/H5; F) β/Desii/H5; G) β/waterfowl/H5; H) β/stall/H5; I) B/mixed/bird/H5; J) β/mixed/environment/H5; K) ϕ/bird/H5; L) ϕ/environment/H5.

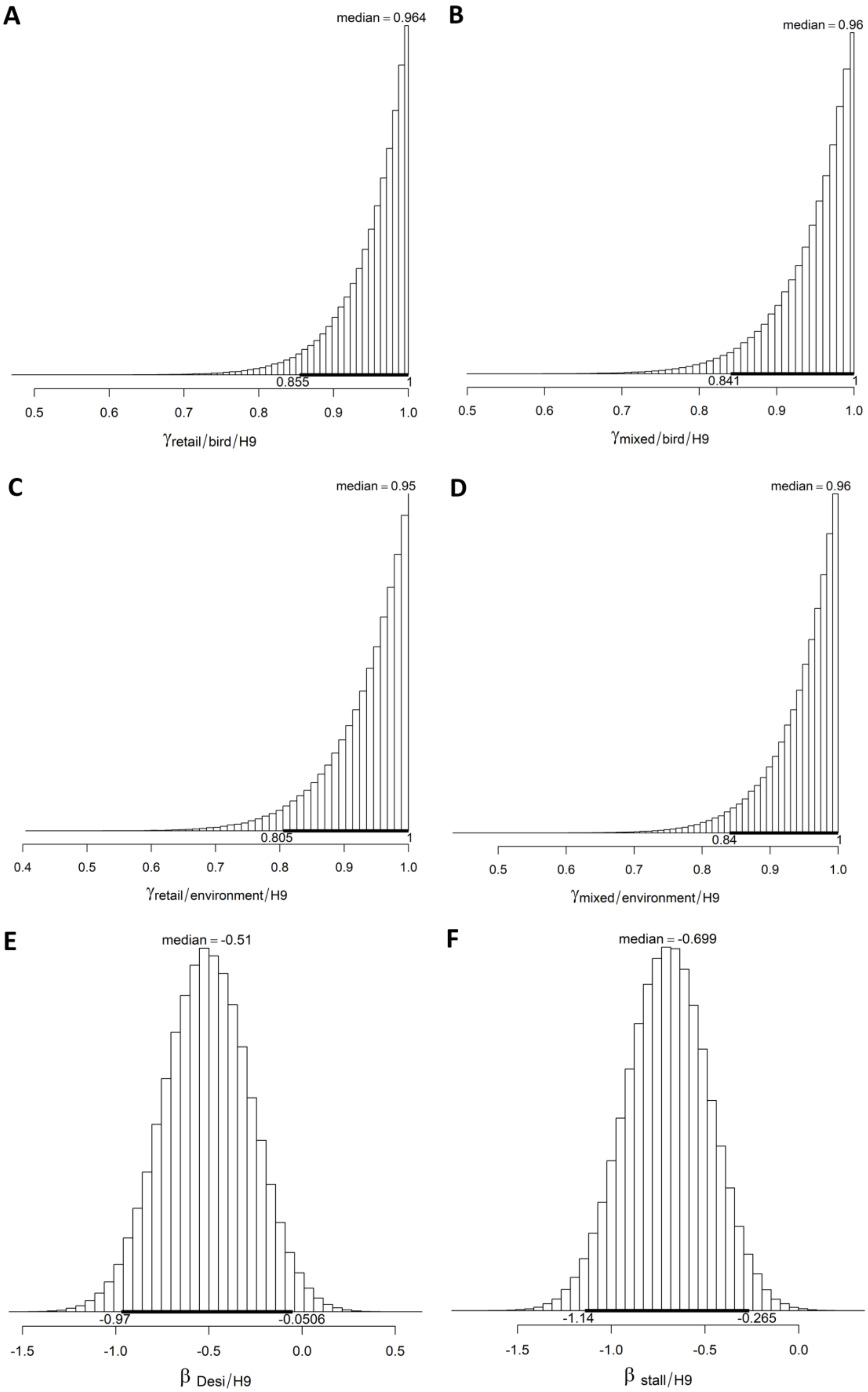
A
median = 0.964
0.855
1
0.5 0.6 0.7 0.8 0.9 1.0
γretail/bird/H9
B
median = 0.96
0.841
1
0.5 0.6 0.7 0.8 0.9 1.0
γmixed/bird/H9
C
median = 0.95
0.805
1
0.4 0.5 0.6 0.7 0.8 0.9 1.0
γretail/environment/H9
D
median = 0.96
0.84
1
0.5 0.6 0.7 0.8 0.9 1.0
γmixed/environment/H9
E
median = -0.51
-0.97
-0.0506
-1.5 -1.0 -0.5 0.0 0.5
β Desi/H9
F
median = -0.699
-1.14
-0.265
-1.5 -1.0 -0.5 0.0
β stall/H9

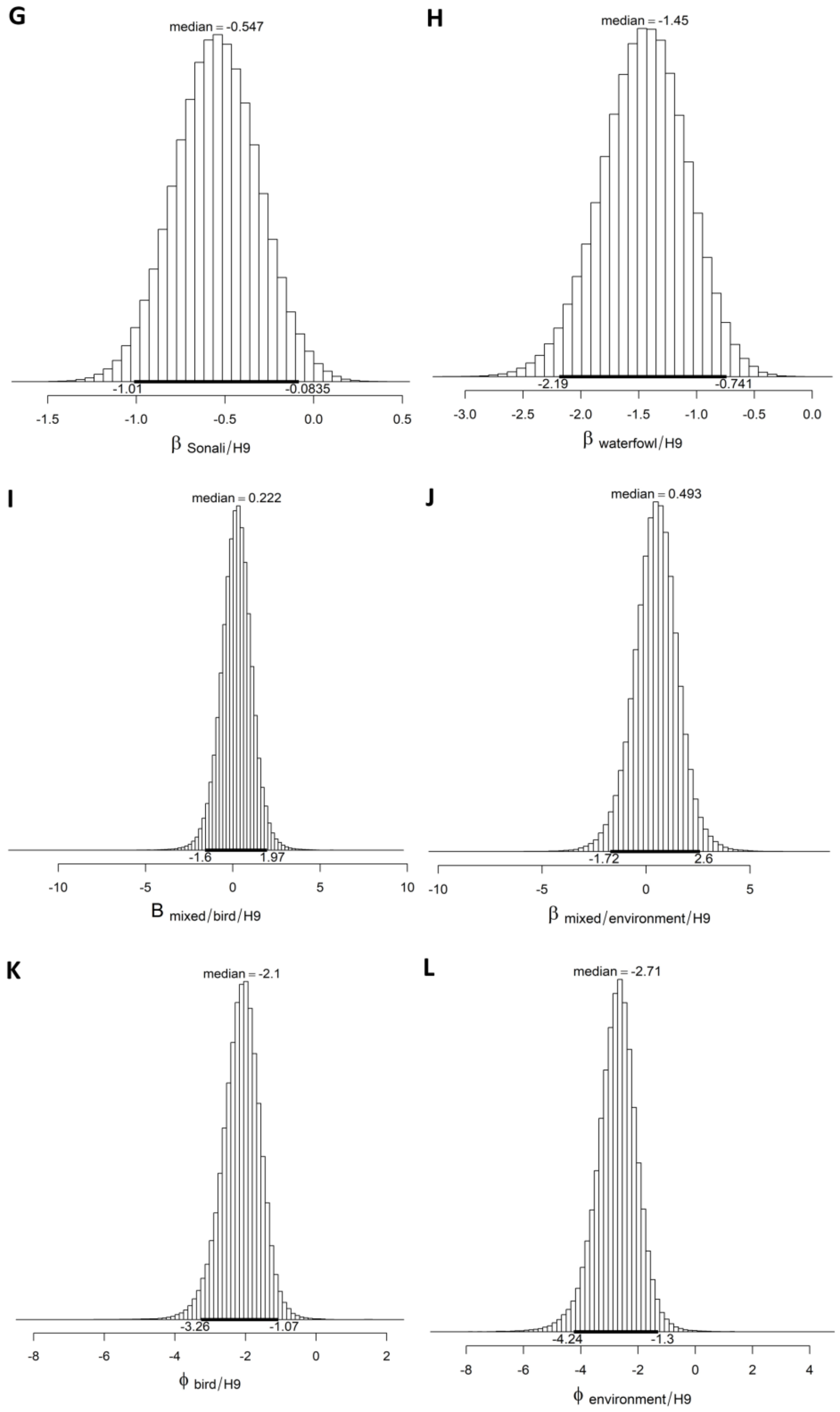

Technical Appendix 2 Figure 3. Posterior distribution of parameters used in models for prevalence of avian influenza A(H9) subtype virus in live bird markets, Bangladesh. Desi, “local” in Bengali, are indigenous chicken breeds raised in backyard farms. Sonali is a cross-breed of the Rhode Island Red

cocks and Fayoumi hens. Values along baselines are medians. Solid horizontal bars indicate 95% high density intervals. Each panel (A–L) shows a different situation that is listed at the bottom of each figure panel. A) γretail/bird/H9; B) γ/mixedl/bird/H9; C) γ/retail/environment/H9; D) γ/mixed/environment/H9; E) β/Desi//H9; F) β/stall/H9; G) β/Sonali/H9; H) β/waterfowl/H9; I) B/mixed/bird/H9; J) β/mixed/environment/H9; K) ϕ/bird/H9; L) ϕ/environment/H9.
